# Influence of Rapid Consolidation on Co-Extruded Additively Manufactured Composites

**DOI:** 10.3390/polym14091838

**Published:** 2022-04-29

**Authors:** Chethan Savandaiah, Stefan Sieberer, Bernhard Plank, Julia Maurer, Georg Steinbichler, Janak Sapkota

**Affiliations:** 1 Bio-Based Composites and Processes, Wood K Plus-Kompetenzzentrum Holz GmbH, 4040 Linz, Austria; 2 Institute for Polymer Injection Moulding and Process Automation, Johannes Kepler University, 4040 Linz, Austria; georg.steinbichler@jku.at; 3 Institute of Structural Lightweight Design, Johannes Kepler University, 4040 Linz, Austria; stefan.sieberer@jku.at; 4 Research Group Computed Tomography, University of Applied Sciences Upper Austria, 4600 Wels, Austria; bernhard.plank@fh-wels.at (B.P.); julia.maurer@fh-wels.at (J.M.); 5 Research Centre of Applied Science and Technology, Tribhuvan University, Kirtipur 44600, Nepal; sapkota.janak@outlook.com

**Keywords:** additive manufacturing, rapid consolidation, composites, carbon fibres

## Abstract

Composite filament co-extrusion (CFC) additive manufacturing (AM) is a bi-matrix rapid fabrication technique that is used to produce highly customisable composite parts. By this method, pre-cured, thermoset-based composite carbon fibre (CCF) is simultaneously extruded along with thermoplastic (TP) binding melt as the matrix. Like additive manufacturing, CFC technology also has inherent challenges which include voids, defects and a reduction in CCF’s volume in the fabricated parts. Nevertheless, CFC AM is an emerging composite processing technology, a highly customisable and user-oriented manufacturing unit. A new TP-based composites processing technique has the potential to be synergised with conventional processing techniques such as injection moulding to produce lightweight composite parts. Thus, CFC AM can be a credible technology to replace unsustainable subtractive manufacturing, if only the defects are minimised and processing reliability is achieved. The main objective of this research is to investigate and reduce internal voids and defects by utilising compression pressing as a rapid consolidation post-processing technique. Post-processing techniques are known to reduce the internal voids in AM-manufactured parts, depending on the TP matrices. Accordingly, the rapid consolidated neat polylactic acid (PLA) TP matrix showed the highest reduction in internal voids, approximately 92%. The PLA and polyamide 6 (PA6) binding matrix were reinforced with short carbon fibre (SCF) and long carbon fibre (LCF), respectively, to compensate for the CCF’s fibre volume reduction. An increase in tensile strength (ca. 12%) and modulus (ca. 30%) was observed in SCF-filled PLA. Furthermore, an approximately 53% increase in tensile strength and a 76% increase in modulus for LCF-reinforced PA6 as the binding matrix was observed. Similar trends were observed in CFC and rapidly consolidated CFC specimens’ flexural properties, resulting due to reduced internal voids.

## 1. Introduction

Composite filament co-extrusion (CFC) additive manufacturing (AM) is a newly developed bi-matrix processing technology. CFC processing combines thermoplastic (TP) filament as a binding matrix and pre-cured thermoset (TS)-based composite carbon fibre (CCF) filament as a reinforcement. The CCF constituents are continuous carbon fibres infused with low viscous epoxy-based thermoset and heat cured to form a filament-like structure for CFC processing. The binding process of TP melt onto the CCF filament is analogous to crosshead extrusion processing of wire and cable coating. This enables the user to select off-the-shelf filled and unfilled binding TP matrix filaments, such as polylactic acid, polyolefin, polyamide, polyimide, et cetera. The carbon fibre volume fraction in CCF is approximately 57 vol.% to 62 vol.% [1,2,3]. However, when combined with a TP binding matrix the volume fraction is further reduced to approximately 18 vol.% to 35 vol.% depending on CCF layer height settings [1,2,4,5].

The incompatibility of TP matrix and carbon fibre in continuous fibre AM, often resulting due to high viscous TP, has been extensively reported by researchers [6,7,8]. The CCF filament matrix is an epoxy-based low viscous TS that conforms to better carbon fibre wetting, and the TS curing process results in the void and defect-free composite filament [1,9]. According to Azarov et al. [1] and Adumitroaie et al. [5], the CCF filament usage in CFC aims to overcome the perceived disadvantages of producing composite parts entirely from TP or TS. The detailed study showed that the CFC technology is aimed at fabricating lattice composites that are often difficult to fabricate by the existing automated technologies [2,9]. Furthermore, researchers concluded that CFC AM, as such, is not suitable to achieve significant improvement in the composite material properties because fabricating defect-free composite parts is challenging [1]. Also, few studies have shown filling the TP matrix [10] and reinforcing it with short [11] and long carbon fibre [12] can increase the mechanical and thermal properties of material-extruded (MEX) AM specimens [13]. Likewise, to compensate for the reduction in CCF fibre volume fraction and increase the flexural properties, the researchers used 20 wt. % short carbon fibre filled polyamide 6 as a binding matrix [14]. The short carbon fibre-filled polyamide 6 as a binding matrix had improved flexural properties compared to the unfilled TP matrix. Furthermore, the researchers found high process-induced voids within the CFC-fabricated specimens for both TP matrices, and reduction in a high degree of voids is elusive due to low compaction in situ consolidation during layered manufacturing.

Van de Werken et al. [15,16], used the hot isotactic pressing (HIP) technique to reduce the process-induced voids by 51% and, improved the flexural strength and interlaminar shear strength of TP-based continuous fibre additive manufactured coupons by 30%–45%. Similarly, Savandaiah et al. [17] performed rapid consolidation on highly anisotropic short and long carbon fibre-reinforced MEX specimens. The post-processing increased the thermal and mechanical properties of MEX specimens comparable to the injection-moulded specimens. The advantage of the rapid consolidation techniques such as compression pressing is the ability to achieve higher densification and reduction in voids between 50% to 75% at a reduced cycle time of less than 30 min [17]. However, in HIP the process duration varies between 1 h to 4 h depending on the type of thermoplastic used in TP-based continuous fibre AM.

Henceforth, the scope of the research was centred on the question of whether the rapid consolidation technique is suitable to achieve better composite material properties in CFC specimens. In this study, the researchers have investigated the influence of polylactic acid (PLA), and polyamide 6 (PA6) as a binding matrix on processing and mechanical properties. Similarly, short carbon fibre-filled PLA and long carbon fibre-reinforced PA6 were studied to quantify the influence of fibre reinforcement on CFC-printed specimens. The tensile and flexural specimens were assessed to evaluate the mechanical properties of CFC and post-processed CFC specimens. Computed tomography (CT) ensured the volume of process-induced voids in the CFC and reduction in post-consolidated specimens.

## 2. Materials and Methods

### 2.1. Materials

PLA homopolymer [18], was bought from Total Corbion, Gorinchem, Netherlands. The particulate carbon fibre without sizing, Tenax HT, was acquired from Teijin Carbon Europe GmbH, Wuppertal, Germany. Film extrusion grade PA6 [19] was purchased from DSM N. V, Geleen, Netherlands. The PLA and PA6 compounding, filament extrusion, and injection moulding (IM) is based on the procedure detailed in the previously published research work [14,17,20]. Short carbon fibre (20 wt.%, length 83 µm) filled PLA was filament-extruded in-house (1.75 mm) and a commercially available long carbon fibre (20 wt.%, length 218 µm) reinforced PA6 filament (1.75 mm) was supplied by Prirevo 3D solutions GmbH, Ried im Traunkreis, Austria. The CCF filament was purchased from Anisoprint SARL, Mondercange, Luxembourg. For simplification, the nomenclature of the samples is presented in Table 1, the thermal and mechanical test data are tabulated in Table 2, and the tensile test data of CCF is given in Table 3.

### 2.2. Preparation of Printed Specimens

**Figure 1:**
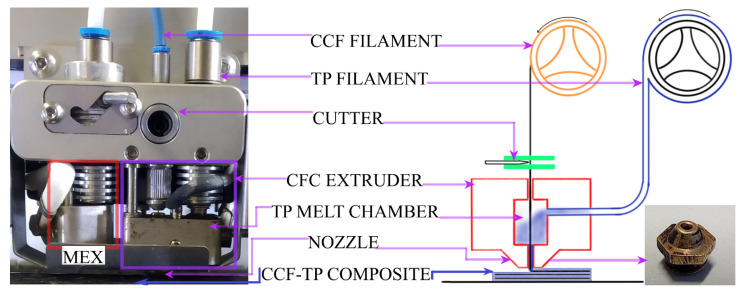
MEX and CFC print head photography and schematics of Composer A4 CFC print head. The photography shows two print heads, a typical MEX print head for plastic printing (red box) and a CFC print head (purple box) for composite printing.

**Figure 2:**
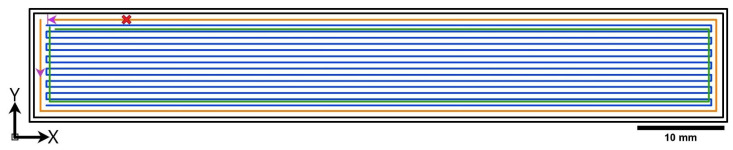
Graphical representation of part configuration generated via slicing software. Black coloured lines are outer thermoplastic perimeters, green coloured line represents micro infill thermoplastic filling by MEX print head, and orange and blue series lines are CCF reinforced perimeter and infill by CFC print head. The purple pointed arrow shows the direction of CCF printing and the pattern “x” marks the symbolic fibre cut operation.

The samples for mechanical characterization were printed in composer A4 CFC printer (Figure 1), with two print heads, manufactured by Anisoprint SARL, Mondercange, Luxembourg. Conventional MEX print head to fabricate parts with unfilled and filled thermoplastic alike, and CFC print head to print CCF with thermoplastic melt as a binder. The instruction sets for the CFC printer were prepared in a proprietary slicing software, AURA (ver. 1.27.3) and the configuration is shown in Figure 2 and summarized in Table 4. The print bed and printed samples were cooled down to nominal room temperature to avoid part distortion and to facilitate the removal. The standard tensile sample dimension 220 × 15 × 3 mm^3^ [21], and standard flexural sample dimension 155 × 13 × 4 mm^3^ [22] were set according to ASTM standards. The sample thickness for rapid consolidation of the flexural specimen was increased 155 × 13 × 4.2 mm^3^, i.e., a single bottom (0.1 mm) and a top layer (0.1 mm) of pure plastic printing was set, to improve the rapid consolidated surface quality and flexural property. Furthermore, after post-consolidation, the specimen thickness was 4 mm, without a major change in final fibre loading. Furthermore, before testing the printed samples were stocked in the standard control cabinet (relative humidity of 50% at 23 °C) for 72 h, as per ASTM D618-21 standard for specimen conditioning [23].

### 2.3. Compression Press Moulding

The compression platen press, LabEcon from Fontijne platen presses and services BV, Delft, Netherlands, was used to rapidly consolidate the printed samples. The rapid consolidation of CFC specimens is based on the procedure detailed in the previously published work [17]. The heating and cooling cycle for each thermoplastic matrix is presented in Table 5. The pressed MEX samples were stored in a controlled cabinet (relative humidity of 50% at 23 °C) for 72 h, as per ASTM D618-21 standard for specimen conditioning [23].

### 2.4. Void Volume Fraction Analyses

X-ray computed tomography (CT) was used to quantify the void content. Therefore, scans were carried out on the Nanotom 180 NF (GE phoenix X-ray, Wunstorf, Germany) laboratory CT device. A molybdenum target and a tube voltage of 60 kV was used for the data acquisition. Similar to Plank et al. [24] and Senck et al. [25], the grey value-based ‘ISO X’ threshold procedure was used for the quantitative evaluation of the void content. This method was already applied to additive manufactured samples [12,26]. The CT scans at a voxel edge length of 7 µm and additional region of interest scans at a voxel edge length of 2 µm were carried out. The latter high-resolution scans were performed on S-PLA-CFC and N-PA6-CFC. Due to the different densities of these materials and different void shapes, two different ISO values were determined. The analyses volume of these high-resolution scans is 3.6 × 3.6 × 3.6 mm³ and was used as a reference for the definition of the appropriate ISO value for void analysis at a voxel edge length of 7 µm. The scans at a voxel edge length of 7 µm provide an analysis volume of the total cross-section (Y-Z section) and allow an approximate length of 13 mm (X-direction) for void analysis.

The void analysis was performed with VGStudio MAX 3.4 (Volume Graphics GmbH, Heidelberg, Germany) software. Moreover, a multistep segmentation of the high-resolution scan with a voxel edge length of 2 µm voxel size was carried out and based on these segmentations the “ISO X” threshold was estimated. For the higher density materials (PLA) an ISO value of 62.75 and for the lower density materials (PA6) an ISO value of 71.4 was defined.

### 2.5. Mechanical Characterisation

For all mechanical testing, a Zwick–Roell servo-hydraulic test rig with a cylinder rated at 25 kN force was used. The test rig was operated by Cubus software in displacement mode. A Zwick–Roell force transducer and the internal displacement sensor of the cylinder were used for measurement. Additionally, a correlated solutions 3D digital image correlation (DIC) system recorded the surface displacement and calculated the surface strains of the specimens during the tests. Post-processing of the DIC data was performed in the software Vic-3D 8.

For tensile testing, MTS 647 hydraulic jaws were used to grip the specimens. Glass fibre composite end tabs were adhesively bonded to the specimens with 3M DP490 epoxy adhesive. Before testing, a speckle pattern was applied to one face of the specimens for DIC measurement. The test procedure followed ASTM standard D3039 [21] for tensile testing of composite laminates with a machine head speed of 2 mm·min^−1^. The test was stopped at the break of the specimens.

For bending testing, 4-point bending testing according to ASTM D6272 [22] was chosen with the load span half of the support span. By this method, the central span of the specimen is in bending only, and no transverse force component exists. This central span is monitored by DIC side on, enabling monitoring of maximum deflection and strains over the thickness. The machine head speed was set at 2 mm·min^−1^ and the maximum displacement was chosen to exceed the strength of the material. For both test cases, stiffness and strength values were calculated according to the respective standards

## 3. Results and Discussion

### 3.1. Void Volume Fraction

**Figure 3:**
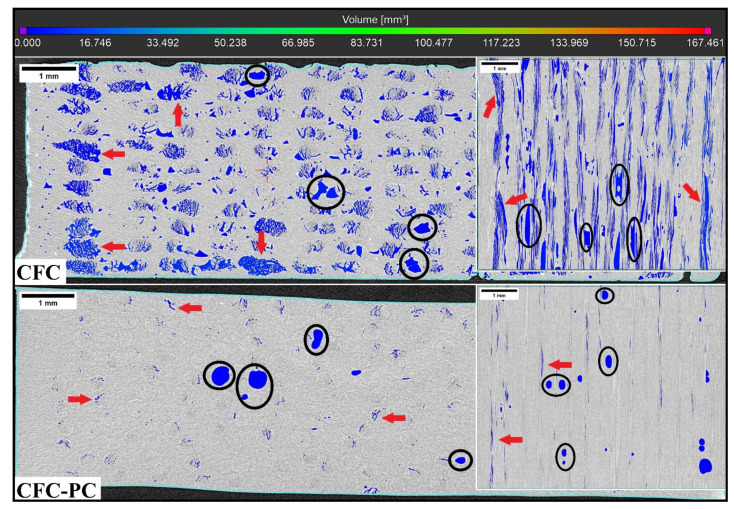
Top midsection CT images (X-Y) of the N-PLA with corresponding side midsection inset views (X-Z). A black circle is indicative of voids due to the thermoplastic matrix and red arrows showed voids within the CFC processed CCF.

The specimen’s void volume fraction is listed in Table 6, and the reference MEX specimen is given along with CFC and post-processed CFC specimens. Voids in the injection-moulded specimens were not detected, as expected, due to high densification, and the void volume fraction in CCF filament was negligible (<0.2 vol.%). In Figure 3, CT-scanned images along with scalar void volume scale of N-PLA specimens are represented with the top midsection view and corresponding insets with the side midsection view. The black circles within N-PLA-CFC and N-PLA-CFC-PC represent voids in the TP matrix, and red arrows indicate voids within pre-cured TS based CCF filament. However, the defects in unprocessed CCF filament were minimal, and, therefore, the voids detected in CFC-processed specimens may be the attributes of epoxy-based TS. According to the dynamic mechanical analysis (DMA) of CCF filament by Adumitroaie et al. [5], these voids correlated to the inhomogeneity in the TS composition and inhomogeneous heat distribution between the CCF’s constituents during AM process as seen in CT images. The high amount of blue-coloured voids seen in N-PLA-CFC, with void volume in the range of 0.01 mm^3^ to 17.0 mm^3^, are distributed throughout the whole sample. The post-processed N-PLA-CFC-PC CT image showed a high reduction in void volume fraction within TP binding matrix as well as CFC processed CCF filament. The total void volume fraction of N-PLA-CFC is 14.0 vol.% and, post-processed N-PLC-CFC-PC is 1.0 vol.%, a 92% reduction in void volume fraction. Also, post-processed S-PLA-CFC-PC and L-PA6-CFC-PC showed a reduction in void volume fraction between 35% to 50% depending on the weighted average fibre length [17].

### 3.2. Tensile Properties

**Figure 4:**
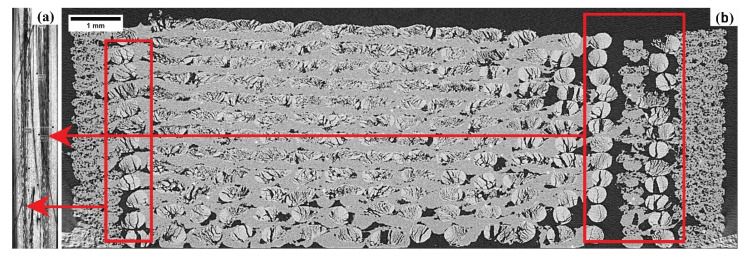
Tensile tested fractured image (**a**) and CT image (X-Y) (**b**) of L-PA6-CFC specimen. The longitudinal failure observed in the tensile tested image (**a**) is due to the defects observed in the CT image is indicated by arrows connected to the defective region (red box) in CT-scanned image (**b**).

All tensile-tested CFC specimens demonstrated longitudinal splitting mode in the defective region shown in Figure 4a. The defective region (red box) in Figure 4b, the area without binding matrix is due to the CFC print head design and pre-set instruction for CFC composite fabrication. The CFC print head flaws are contributing to defective processing and increased unreliability in the established processing settings, specifically in fibre reinforced binding matrix [27]. Furthermore, L-PA6 binding matrix has the highest void composition compared to the short fibre-filled TP and neat TP binding matrix (Table 6). Comparable results were observed earlier [12,17] showing a correlation between increased fibre lengths and increasing void volume fraction in the MEX specimens.

**Figure 5:**
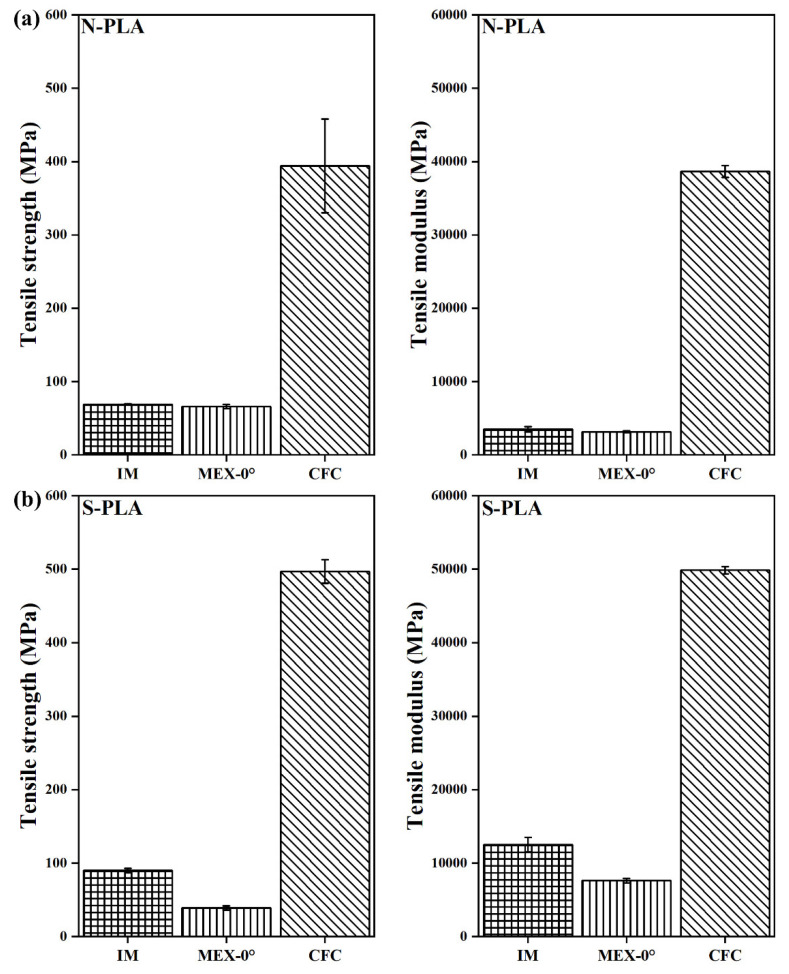
Tensile test data of PLA with a corresponding error bar. Where, N-PLA is neat PLA and S-PLA is short carbon fibre filled PLA. The corresponding IM, MEX-0°, and CFC are injection moulded, 3D printed and composite filament co-extruded 3D printed tensile tested specimens, respectively.

**Figure 6:**
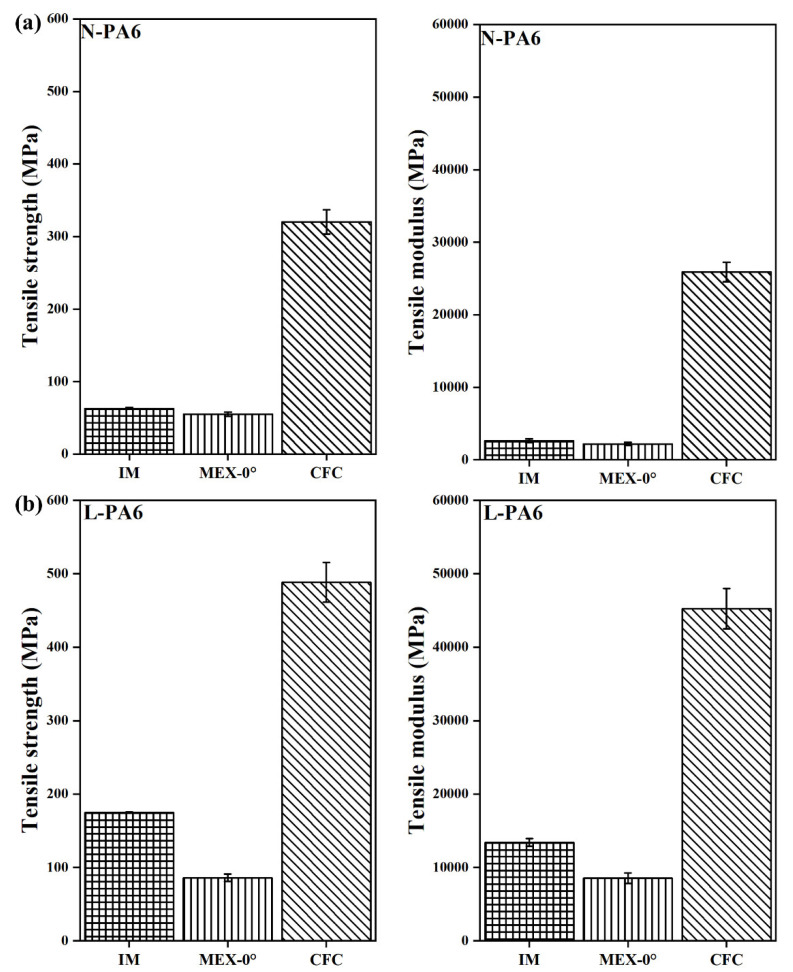
Tensile test data of PA6 with a corresponding error bar. Where, N-PA6 is neat PA6 and L PA6 is long carbon fibre reinforced PA6. The corresponding IM, MEX-0°, and CFC are injection moulded, 3D printed and composite filament co-extruded 3D printed tensile tested specimens, respectively.

Figure 5a,b shows comparative tensile test results of N-PLA-CFC and S-PLA-CFC along with injection-moulded and MEX AM specimens as reference for binding matrix. Similarly, Figure 6a,b showed tensile test results of N-PA6-CFC and L-PA6-CFC, respectively. An increase in tensile strength is observed for S-PLA-CFC (497 ± 16 MPa) compared to N-PLA-CFC (394 ± 64 MPa) and a 30% increase in S-PLA-CFC (49,850 ± 505 MPa) modulus. Also, a large standard deviation in CFC processed specimens is indicative of previously discussed defects associated with CFC AM processing.

Figure 6a shows tensile test results of N-PA6-CFC along with tensile test results of L-PA6-CFC in Figure 6b. A 53% increase in L-PA6-CFC tensile strength (TS) (488 ± 27 MPa) compared to N-PA6-CFC (320 ± 17 MPa) and a 76% increase in tensile modulus (TM) in CFC processed L-PA6 (45240 ± 2740 MPa). The increased weighted average length contributes positively to the mechanical performance of CFC processed L-PA6. However, the total fibre volume fraction in CCF decreased drastically and contributed to the loss in tensile properties, approximately 67% and 80% in tensile modulus and strength, respectively. Defects detected by CT such as CCF splitting-spreading, and therefore, CCF is no longer consolidated and contributed to early failure as shown in Figure 4a.

### 3.3. Flexural Properties

Flexural strength is purely based on the TP binding matrix and, therefore, it is essential to perform the rapid consolidation study on the flexural standard specimens. The 4-point flexural specimens showed elastic response followed by the plastic collapse in one or more sections in the central span. Some influences of the load application rollers were visible, potentially from high contact stresses and subsequent indentation of the material. In situ detection of fracture modes by the DIC system is difficult because of the limited number of measurements on the thin specimen side.

**Figure 7:**
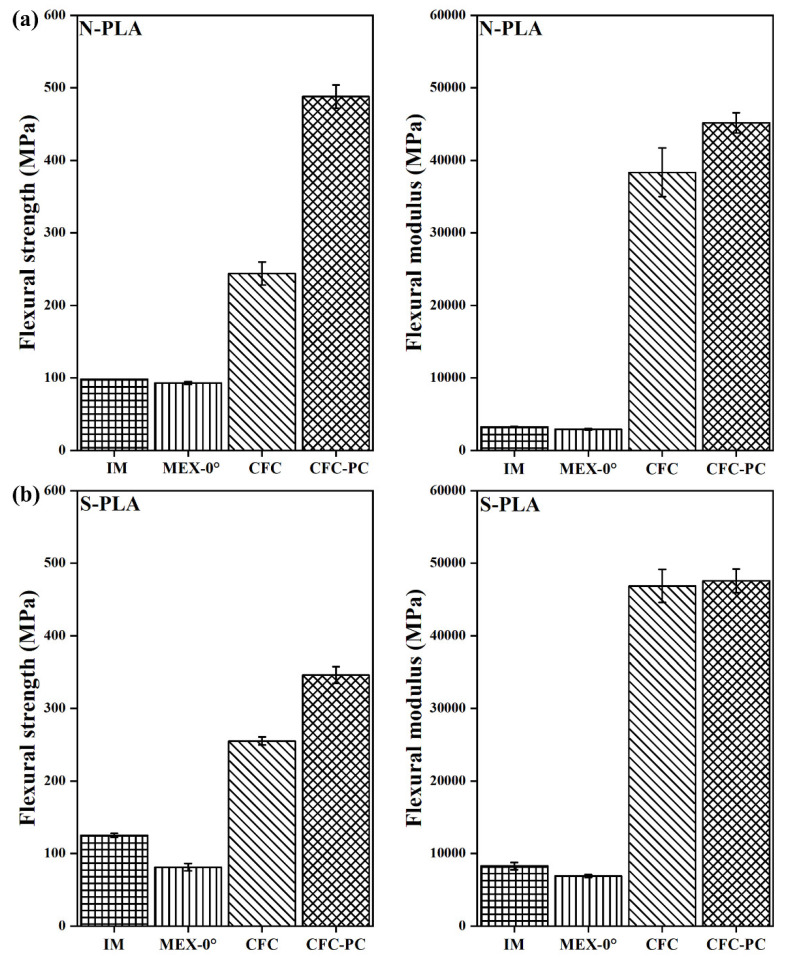
Flexural test data of PLA with a corresponding error bar. Where, N-PLA is neat PLA and S-PLA is short carbon fibre filled PLA. The corresponding IM, MEX-0°, CFC, and CFC-PC are injection moulded, 3D printed, composite filament co-extruded, and post-processed composite filament co-extruded 3D printed 4-pointing beding tested specimens, respectively.

In Figure 7a, the post-processed N-PLA-CFC-PC (488 ± 15 MPa) specimen showed increased flexural strength (FS) by approximately 50% compared to N-PLA-CFC (244 ± 16 MPa). Furthermore, there was an 11% increase in flexural modulus (FM) after post-processing for N-PLA-CFC-PC. On the contrary, the influence of short fibre filling on PLA as a binding matrix had minimal effect on the FM but an approximately 37% increase in FS after post-processing. Furthermore, N-PLA-CFC-PC coupons with the highest reduction in void volume fraction (Table 6) showed similar FS and FM (45180 ± 1390 MPa) compared to S-PLA-CFC (FS= 255 ± 5.5 MPa and FM= 46850 ± 2280 MPa) and S-PLA-CFC-PC (FS= 346 ± 11.5 MPa and FM= 47580 ± 1640 MPa).

**Figure 8:**
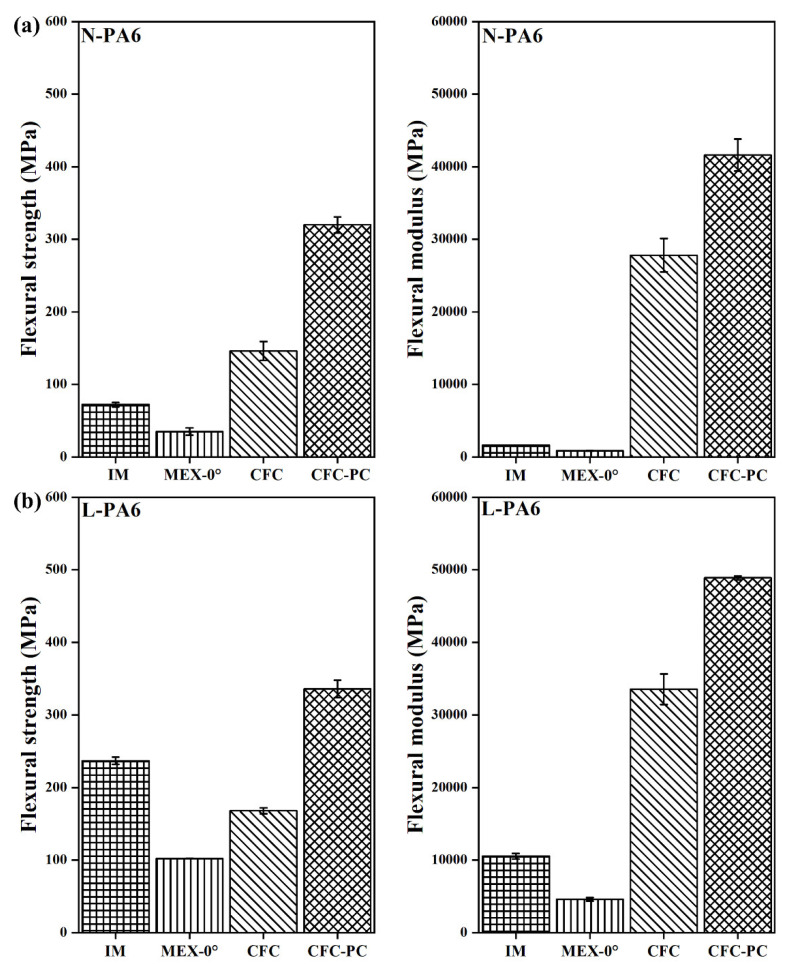
Flexural test data of PA6 with a corresponding error bar. Where, N-PA6 is neat PA6 and L PA6 is long carbon fibre reinforced PA6. The corresponding IM, MEX-0°, CFC, and CFC-PC are injection moulded, 3D printed, composite filament co-extruded, and post-processed composite filament co-extruded 3D printed 4-pointing beding tested specimens, respectively.

In Figure 8a, post-processed N-PA6-CFC-PC (FS= 320 ± 11.5 MPa and FM = 41,600 ± 2200 MPa) demonstrates flexural properties increase by two-fold compared to N-PA6-CFC. In contrast, L-PA6-CFC showed FS lower than referred injection-moulded (L-PA-IM) binding TP matrix (Figure 8b). The cause for flexural underperformance of CCF reinforced L-PA6-CFC is discussed in the void fraction analysis section as it can be correlated to the large number of defects observed in Figure 4, and similarly tabulated in Table 6. Moreover, after a reduction in void volume fraction by approximately 38%, the FS and FM of post-processed L-PA6-CFC-PC increased.

**Figure 9:**
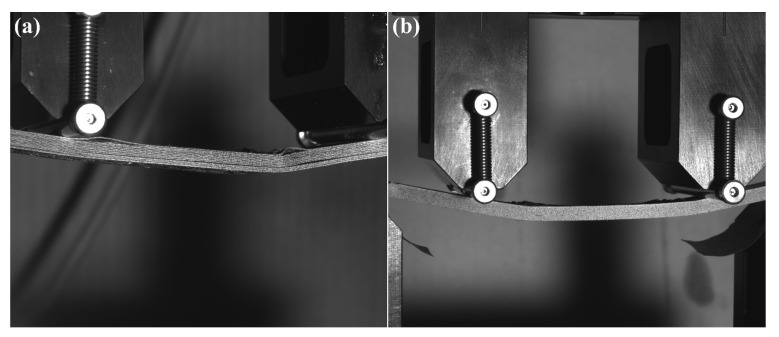
Specimen Nr. 3 of N-PA6-CFC showing delamination (**a**) and specimen Nr. 2 of N-PA6-CFC-PC showing buckling with little delamination at failure (**b**).

Figure 9 shows N-PA6 specimens as-printed and post-consolidated after failure. The as-printed specimens showed delamination, whereas the post-consolidation eliminates the tendency to delaminate. In contrast, N-PLA-CFC specimens exhibited plastic deformation without visible large-scale delamination. For S-PLA-CFC and L-PA6-CFC, there was plastic deformation (plastic knee) without visible delamination. Because of the large deflections, buckling of the fibres in the printed layers was visible for most specimens in the top layer where the largest compressive strains were present. Furthermore, in a few instances, the contact of the top supports led to peeling of the top layer under large deformations, however, these damage modes occurred after passing the bending strength.

The S-PLA based binding matrix showed better tensile and flexural properties compared to L-PA6 as a binding matrix, even though the weight average fibre length of carbon fibre in L-PA6 were longer. This can be attributed to the process-induced annealing effect in the PLA matrix due to high bed temperature, maintained above the glass transition temperature [20,28].

## 4. Conclusions

In summary, the results show that rapid consolidation as a post-processing technique is highly beneficial, given the amount of defects detected in the CFC processed specimens. Void volumetric fraction reduction between 50% and 90% determined by CT scans was achieved, resulting in a 10% to 80% increase in flexural properties. The next valid step is to reduce the voids by applying more compaction pressure through the nozzle on the discharged fibre strands. This would be achieved by slightly reducing the layer height and thus increasing the fibre volume fraction in composite parts. This can further improve the mechanical properties of the rapid consolidated CFC parts. The compliance of upscaling the composite CFC AM to serial production is difficult due to inherent process-induced voids, and, therefore resulting ininconsistent part’s quality. Hence, in this article, the researchers have shown the advantages of rapid consolidation as a post-processing methodology to overcome the perceived disadvantage in composite CFC AM.

## Figures and Tables

**Table 1:** Material nomenclature.

Material Description	Nomenclature
Neat PLA	N-PLA
Injection-moulded neat PLA	N-PLA-IM
Neat PLA material extruded with a raster angle of 0°	N-PLA-MEX-0°
Neat PLA as a binding matrix in CFC	N-PLA-CFC
Post-consolidated neat PLA as a binding matrix in CFC	N-PLA-CFC-PC
Short carbon fibre-filled PLA	S-PLA
Injection-moulded short carbon fibre-filled PLA	S-PLA-IM
Short carbon fibre-filled PLA material extruded with a raster angle of 0°	S-PLA-MEX-0°
Short carbon fibre-filled PLA as a binding matrix in CFC	S-PLA-CFC
Post-consolidated short carbon fibre-filled PLA as a binding matrix in CFC	S-PLA-CFC-PC
Neat PA6	N-PA6
Injection-moulded neat PA6	N-PA6-IM
Neat PA6 material extruded with a raster angle of 0°	N-PA6-MEX-0°
Neat PA6 as a binding matrix in CFC	N-PA6-CFC
Post-consolidated neat PA6 as a binding matrix in CFC	N-PA6-CFC-PC
Long carbon fibre-reinforced PA6	L-PA6
Injection-moulded long carbon fibre-reinforced PA6	L-PA6-IM
Long carbon fibre reinforced PA6 material extruded with a raster angle of 0°	L-PA6-MEX-0°
Long carbon fibre-reinforced PA6 as a binding matrix in CFC	L-PA6-CFC
Post-consolidated long carbon fibre-reinforced PA6 as a binding matrix in CFC	L-PA6-CFC-PC

**Table 2:** Injection-moulded material thermal test data. Melting temperature (MT), heat deflection temperature (HDT).

Properties	Test	Unit	N-PLA	S-PLA	N-PA6	L-PA6
MT	ISO-11357-1:2016	°C	170–175	170–175	220–225	220–225
HDT	EN ISO 75-HDT A	°C	58.0 ± 0.1	59.0 ± 0.2	48.0 ± 0.3	-- *
HDT	EN ISO 75-HDT C	°C	--	--	--	133.0 ± 3.0

* No maximum deflection at maximum temperature (testing machine limit at 200 °C).

**Table 3:** CCF tensile test data. For the tensile test, 100 mm of CCF was taken directly from the spool, tabs were glued onto each end for gripping and to reduce damage to CCF during clamping and testing.

Properties	Unit	Value
Diameter	mm	0.36
Tensile strength	MPa	2224 ± 283
Young’s modulus	MPa	130000 ± 9000
Elongation at break	%	1.6 ± 0.2
Fibre volume fraction	%	57

**Table 4:** Important printing settings.

Parameter	Unit	PLA	PA6
CFC nozzle temperature	°C	225	255
MEX nozzle temperature	°C	220	250
CFC TP flow multiplier	--	0.95	1.05
CFC layer height	mm	0.36	0.36
CFC extrusion width	mm	0.75	0.75
MEX TP flow multiplier	--	0.90	1
MEX layer height	mm	0.12	0.12
MEX extrusion width co-efficient	--	1	1.05
Bed temperature	°C	80	95
TP perimeter count	--	2	2
Inner CCF perimeter count	--	1	1
CCF infill pattern	--	Solid	Solid
CCF infill angle	°	0	0
MEX print speed	mm·s^−1^	60	60
CFC print speed	mm·s^−1^	10	10

**Table 5:** Compression pressing process conditions.

Setting	PLA	PA6
Set temperature	180 °C	220
1st cooling cycle	cool down to 70 °C at 50 °C·min^−1^	cool down to 150 °C at 50 °C·min^−1^
2nd cooling cycle	cool down to 23 °C at 5 °C·min^−1^	cool down to 50 °C at 5 °C·min^−1^

**Table 6:** Influence of rapid consolidation on void reduction in CFC specimen. For reference, the void volume fraction of the corresponding MEX specimen with a raster angle of 0° is reported.

	Void (Vol. %)		
Material	MEX-0°	CFC	CFC-PC
N-PLA	12.2 *	14.0	1.0
S-PLA	15.9 *	16.3	8.6
N-PA6	14.3 *	16.8 *	8.3
L-PA6	27.0 *	29.2	18.2

* Void volume fraction was taken from previously published research work [14,17,20].

## Data Availability

Further data is available on request from the authors.
